# Sedative and Immunosuppressive Effects of Dexmedetomidine in Transplantation

**DOI:** 10.3390/ph14080825

**Published:** 2021-08-22

**Authors:** Chen-Fang Lee, Chih-Hsien Cheng, Hao-Chien Hung, Jin-Chiao Lee, Yu-Chiao Wang, Tsung-Han Wu, Ting-Jung Wu, Hong-Shiue Chou, Kun-Ming Chan, Wei-Chen Lee

**Affiliations:** 1 Department of Liver and Transplantation Surgery, Chang-Gung Memorial Hospital at Linkou, Taoyuan City 333, Taiwan; chengcchj@gmail.com (C.-H.C.); mp0616@cgmh.org.tw (H.-C.H.); chiaopee@gmail.com (J.-C.L.); awuang726@gmail.com (Y.-C.W.); wutsunghan@gmail.com (T.-H.W.); wutj5056@gmail.com (T.-J.W.); chouhs@cgmh.org.tw (H.-S.C.); chankunming@cgmh.org.tw (K.-M.C.); weichen@cgmh.org.tw (W.-C.L.); 2 College of Medicine, Chang-Gung University, Taoyuan City 333, Taiwan

**Keywords:** delirium, tolerance, sedation, liver transplant, outcome

## Abstract

Dexmedetomidine, an α2-adrenergic receptor agonist, is used as an anti-anxiety medication. It exerts a cholinergic effect, thereby reducing the release of tumor necrosis factor alpha (TNF-α). We hypothesized that the use of dexmedetomidine as a sedative agent in transplantation would also protect allografts. We examined our patients who underwent living donor liver transplantation. Subsequently, we generated a series of mouse models to investigate the effect of dexmedetomidine on sedation-based tolerance post transplantation. A total of 49 liver recipients were enrolled in this study, of which 23 (47%) were administered dexmedetomidine through 24 h infusion on postoperative day 1. A trend toward the improvement of hepatocyte injury along with better liver function was observed in the dexmedetomidine-treated group during the first postoperative week. In animal models, dexmedetomidine inhibited the proliferation of CD4^+^ and CD8^+^ T cells and TNF-α production in a dose-dependent manner. We used dexmedetomidine to treat skin-transplanted mice and observed a significantly prolonged graft survival in mice that were administered a higher dose of dexmedetomidine. Our results revealed that dexmedetomidine exerts a dual effect of sedation and immunosuppression. This light-sedation approach will not only make patients calmer in the intensive care unit but also protect allografts from injury.

## 1. Introduction

Patients who undergo liver transplantation (LT) are at a high risk of delirium in the intensive care unit (ICU) [1,2]. The reported frequency of delirium in these recipients ranges from 12.7% to 47% [2]. Since delirium causes more complications and leads to longer stays in the ICU, it is important to identify patients who are at a higher risk and provide adequate sedation.

Dexmedetomidine, an α_2_-adrenergic receptor agonist, is used as an anti-anxiety medication and analgesic [3]. Since its sedative effect is mediated via the non-γ-aminobutyric acid (GABA) pathway, dexmedetomidine is thought to be less delirium-inducing and induce a more natural sleep cycle without the risk of respiratory depression [4]. The use of dexmedetomidine to prevent delirium in patients with LT or critical illness has been extensively recommended [1,5,6,7].

Some studies have reported that the electrical stimulation of the vagus nerve in animals activates choline acetyltransferase-positive T cells to secrete acetylcholine in the spleen and other tissues [8,9,10]. They also revealed that the stimulation of the vagus nerve inhibits TNF-α and attenuates disease severity in autoimmune disorders, such as rheumatoid arthritis and Crohn’s disease [9,10]. Interestingly, recent studies have also demonstrated that dexmedetomidine exerts a cholinergic effect that helps reduce the release of the pro-inflammatory protein TNF-α [11,12]. It has been shown to exhibit anti-inflammatory properties through promoting macrophage phagocytosis and bactericidal activity and reducing the levels of TNF-α, IL-1, and IL-6 in patients with sepsis [11]. These immunological findings have attracted researchers’ interest in assessing the effects of dexmedetomidine in major operations or transplantation. Indeed, the intraoperative infusion of dexmedetomidine has been shown to reduce ischemia–reperfusion injury during hepatectomy and liver transplantation [13,14], indicating its ability to reduce inflammation and immune modulation during operations. However, the impact of dexmedetomidine on LT in the early post-transplant period or long-term graft survival is unknown.

In this study, we hypothesized that the application of dexmedetomidine as a sedative agent post-transplantation may also help avoid allograft rejection. To analyze the anti-inflammatory and immunosuppressive effects of the post-transplant infusion of dexmedetomidine, we reviewed our data from LT recipients and designed a series of animal experiments, along with the generation of a mouse model of skin transplant to investigate the efficacy of dexmedetomidine treatment on sedation-based tolerance and the possible underlying mechanisms.

## 2. Results

### 2.1. Dexmedetomidine Reduces Reperfusion Injury and Prevents Allograft Rejection in Human Liver Transplantation

Since 2016, our institute has prescribed dexmedetomidine to prevent pain and aggravated delirium to patients after LT. We did not use dexmedetomidine during anesthesia but administered it through continuous infusion (0.2 mcg/kg/h) to the recipients upon arrival to the ICU after operation unless they were hemodynamically unstable. The maximum infusion time was 24 h. The infusion of dexmedetomidine was terminated without restarting in the event of bradycardia, hypotension, or severe allergic reactions. Sedation levels were assessed using the Richmond Agitation Sedation Scale (RASS) [15]. The infusion of dexmedetomidine was titrated to achieve sedation, which was primarily light (RASS score of 0–2). Patients who underwent LT before 2016 without the use of dexmedetomidine were included in the control group. Finally, a total of 49 adult-to-adult living donor liver transplantations performed between 2013 and 2019 were retrospectively enrolled in this study, of which 23 (47%) were sedated using dexmedetomidine and 26 (53%) were assigned to the control group. The comparison of demographic and in-hospital characteristics between the two groups is summarized in Table 1. There was no difference in the recipients’ age and sex, donors’ age and sex, disease severity, ascites, or graft-to-recipient weight ratio between the two groups. In the dexmedetomidine and control groups, most of the patients received the right lobe from their donors (95.7% vs. 92.3%, *p* > 0.999), and the major cause of liver cirrhosis was identified as hepatitis B (56.5% vs. 38.5%, *p* = 0.206). The proportions of hepatocellular carcinoma of two groups were 39.1% and 34.6%, *p* = 0.744. We assessed the reduction rates of aspartate aminotransferase (AST) and alanine aminotransferase (ALT) on postoperative days (POD) 2 and 4. Although not statistically significant, a trend towards the decrease in liver transaminase levels was observed in the dexmedetomidine group. The patients in the dexmedetomidine group exhibited more reduction rates of AST and ALT than those in the control group on POD 2 (AST: 32.75% ± 4.23% vs. 29.74% ± 6.44%, *p* = 0.873; ALT: 0.14% ± 0.47% vs. 0.016% ± 0.01%, *p* = 0.317.) and POD 4 (AST: 0.637% ± 0.045% vs. 0.636% ± 0.081%, *p* = 0.873; ALT: 0.39% ± 0.08% vs. 0.25% ± 0.01%, *p* = 0.087). The dexmedetomidine group also exhibited better liver function on POD 7: total bilirubin (4.93 ± 1.45 vs. 5.55 ± 1.5 mg/dL, *p* = 0.96), and international normalized ratio (INR) (1.3 ± 0.036 vs. 1.2332 ± 0.034, *p* = 0.148). These results indicate that dexmedetomidine has the potential to reduce liver injury.

**Figure 1:**
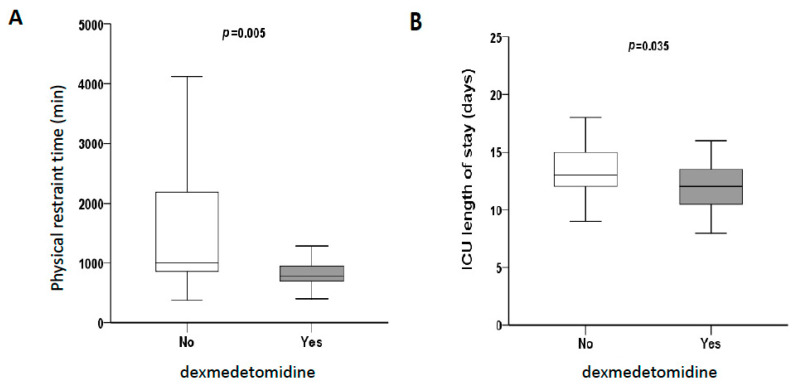
The dexmedetomidine group patients exhibited higher compliance and a shorter length of stay in the intensive care unit (ICU). (**A**) Total physical restraint time for patients in the dexmedetomidine group was significantly decreased (780 ± 273 vs. 1005 ± 1348 min, *p* = 0.005). (**B**) The length of stay in the ICU was also significantly decreased in the dexmedetomidine group (12 ± 4 vs. 13 ± 3 days, *p* = 0.035).

**Figure 2:**
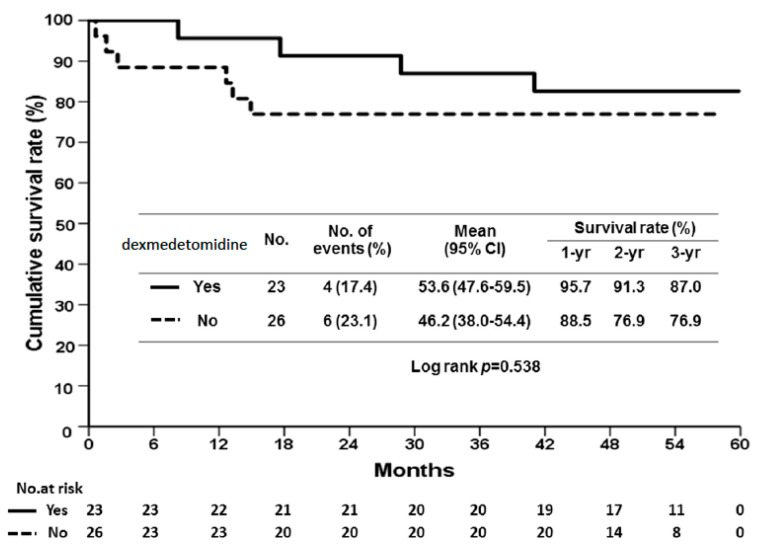
Comparisons of overall survival (OS) between dexmedetomidine and control groups. There was no significant difference in the overall survival (OS) between the two groups. The 1-, 2-, and 3-year OS rates were 95.7% vs. 88.5%, 91.4% vs. 76.9%, and 87% vs. 76.9% (*p* = 0.538, log-rank analysis) in the dexmedetomidine and control groups, respectively.

Regarding the sedative effect, we used the confusion assessment method (CAM) [16] for the assessment of delirium; however, only a few delirium cases were identified in our cohort. Therefore, we recorded the total physical restraint time as an indicator of patients’ levels of consciousness and compliance. Physical restraints were used to maintain the patients’ device and therapy postoperatively. Once the patients were judged to be cooperative without delirium, the restraints were removed. The total physical restraint time for patients in the dexmedetomidine group was found to be significantly decreased (780 ± 273 vs. 1005 ± 1348 min, *p* = 0.005, Figure 1A), which means that dexmedetomidine made our patients calmer and minimized the reliance on restraint in critical care settings. The length of stay in the ICU was also significantly reduced in the dexmedetomidine group (12 ± 4 vs. 13 ± 3 days, *p* = 0.035, Figure 1B), indicating more uneventful courses. We found no significant difference in the overall survival (OS) between the two groups. The 1-, 2-, and 3-year OS rates were 95.7% vs. 88.5%, 91.4% vs. 76.9%, and 87% vs. 76.9% in the dexmedetomidine and control groups, respectively (*p* = 0.538, Figure 2).

Based on the above-mentioned results, we hypothesized that dexmedetomidine may exhibit the potential to not only prevent delirium but also to improve hepatocyte injury after LT. However, the 24 h infusion of dexmedetomidine may not be sufficient to achieve significant differences in avoiding reperfusion injury and allograft rejection. Therefore, we then used a mouse full-thickness skin transplant model to better characterize the effects of dexmedetomidine on sedation-based tolerance and dissect the possible underlying mechanisms.

### 2.2. Dexmedetomidine Abrogates Activation-Induced T-Cell Proliferation and Cytokine Production

**Figure 3:**
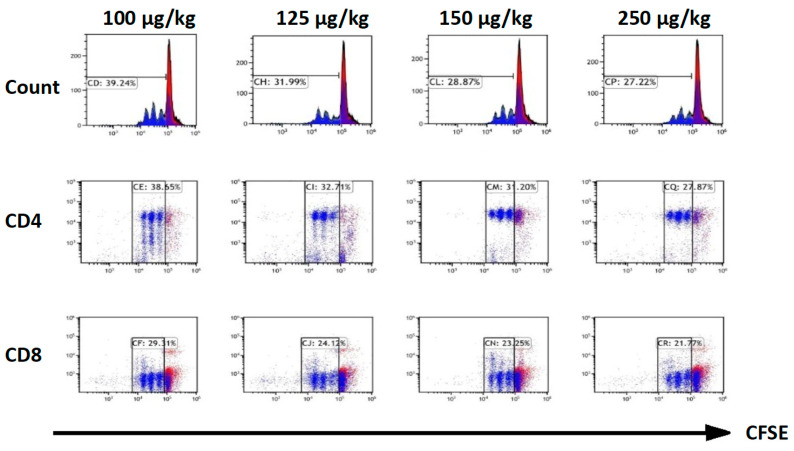
Dexmedetomidine abrogates activation-induced T-cell proliferation. Naïve splenocytes from wide-type C57BL/6 mice labeled with cell proliferation dye carboxyfluorescein succinimidyl ester (CFSE) were stimulated with anti-CD3 (1 µg/mL) after the treatment with dexmedetomidine intraperitoneally with titration concentrations twice a day for 48 h. The proliferation of CD4^+^/CD8^+^ was assessed upon the dilution of CFSE. The results of these titration studies demonstrated that dexmedetomidine inhibited CD4^+^ and CD8^+^ T-cell proliferation in a dose-dependent manner. Data are representative of three independent experiments.

First, we sought to determine the effect of dexmedetomidine on CD4^+^ and CD8^+^ T-cell proliferation. To this end, a titration dose (from 100 to 250 µg/kg) of dexmedetomidine was injected into the peritoneal cavity of C57BL/6 mice twice a day. Three days later, naïve T cells were harvested from the spleen and stimulated with anti-CD3 (1 µg/mL) in the culture medium for 48 h. The proliferation of CD4^+^/CD8^+^ cells was assessed by the dilution of cell proliferation dye carboxyfluorescein succinimidyl ester (CFSE). The results of these titration studies demonstrated that dexmedetomidine markedly inhibited CD4^+^ and CD8^+^ T-cell proliferation in a dose-dependent manner (Figure 3)

**Figure 4:**
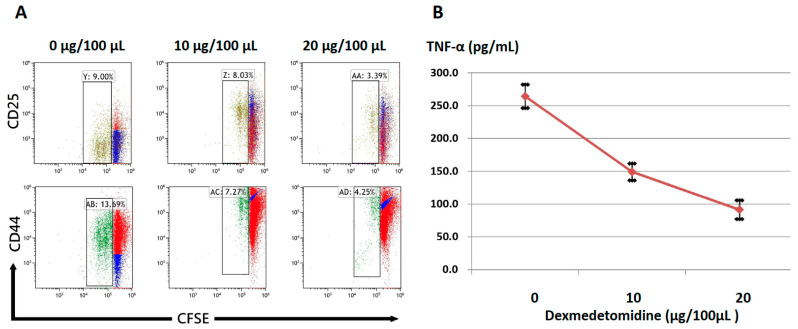
Dexmedetomidine inhibits T-cell activation and cytokine production. Splenocytes from wide-type C57BL/6 mice were stimulated with anti-CD3 (1 µg/mL) and cultured in the presence of dexmedetomidine with titration concentrations. (**A**) The expression of the activation markers, CD44 and CD25, in viable T cells at 48 h. Data are representative of two independent experiments. (**B**) TNF-α secretion in the supernatant was examined using enzyme-linked immunosorbent assay (ELISA). These data suggest that dexmedetomidine inactivates T cells. Data are shown as mean ± SEM of three independent samples.

Next, an in vitro model was used to further investigate the suppressive effect of dexmedetomidine on T-cell activation; splenocytes from wild-type C57BL/6 mice were stimulated with anti-CD3 (1 µg/mL) and cultured in the presence of titration concentrations of dexmedetomidine for 48 h. The expression of the activation markers, CD44 and CD25, in CFSE-labeled T cells at 48 h was evaluated using flow cytometry analysis. We found that dexmedetomidine suppressed the expression of both CD44 and CD25 and inhibited T-cell proliferation in a dose-dependent manner (Figure 4A). To test the ability of dexmedetomidine to mitigate cytokine production, the TNF-α concentration in the supernatant of cell cultures was evaluated using enzyme-linked immunosorbent assay (ELISA). As a result, the marked inhibition of TNF-α production was observed, as shown in Figure 4B. Notably, the decrease in CD4^+^ and CD8^+^ cell function was not due to a decrease in cell viability (data not shown). Together, these data suggest that dexmedetomidine potently inhibits T-cell activation and proliferation.

### 2.3. Dexmedetomidine Promotes Allograft Acceptance in Mouse Models of Skin Transplantation

**Figure 5:**
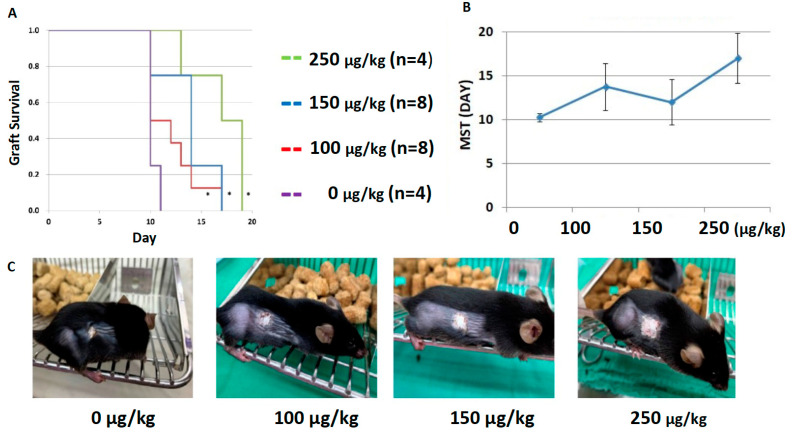
Dexmedetomidine promotes allograft acceptance in mouse models of skin transplantation. (**A**,**B**) Dexmedetomidine was used to treat the skin-transplanted C57BL/6 mice intraperitoneally twice a day. There was a significant increase in the median survival time (MST) compared to those that received no treatment (* all *p* < 0.05, log-rank analysis). (**C**) Representative photographs of the skin grafts at postoperative day 11. Data are representative of two independent experiments.

After demonstrating the ability of dexmedetomidine to inhibit T-cell proliferation and activation, we next investigated whether it could prolong the allograft survival using a fully MHC-mismatched skin transplantation model involving C57BL/6 mice that received allogenic skin grafts from BALB/c mice. We employed dexmedetomidine in titration doses (from 0 to 250 µg/kg) intraperitoneally twice a day to treat the skin-transplanted mice and examined the median survival time (MST) of skin grafts. We found significantly prolonged graft survival in mice that received a higher dose of dexmedetomidine compared to those that received a lower dose or no treatment (all *p* < 0.05, Figure 5A,B). The grafts were monitored daily, and the macroscopic appearance of skin grafts in the higher dose group exhibited less tissue destruction and much healthier alignment. The representative photographs of the skin grafts at POD 11 are presented in Figure 5C. These data further support the idea that sedation-based immunosuppression can be effectively applied to prolong allograft survival.

## 3. Discussion

In this study, we designed a light-sedation experiment to prevent allograft rejection using dexmedetomidine that inhibits T-cell proliferation and activation. Specifically, the significance and major findings of our study involved the targeting of cholinergic anti-inflammatory pathways using dexmedetomidine as an effective agent to mitigate CD4^+^/CD8^+^ T-cell proliferation and activation, thereby avoiding acute allograft rejection. This study is based on the findings that dexmedetomidine, an α_2_-agonist, reduces the release of TNF-α and prevents both delirium and hepatocyte injury in human liver transplantation [1,13]. Although a postoperative 24 h continuous infusion of dexmedetomidine did not result in significant differences in the incidence of rejection and overall survival, the dexmedetomidine group patients exhibited higher compliance and reduced length of stay in the ICU. Patients in the dexmedetomidine group also exhibited higher reduction rates in the liver transaminase levels than those in the control group, thereby indicating the ability of dexmedetomidine to exert protective effects against inflammation and liver allograft rejection. To gain a better understanding of this therapeutic potential, a fully MHC-mismatched mouse skin transplantation model was used to assess the efficacy of dexmedetomidine on sedation-based tolerance. Through our animal model, we demonstrated that the continuous administration of dexmedetomidine may inhibit T-cell activation and result in prolonged skin allograft survival.

The link between sedation and immunity is now being recognized. Recent advances in the interaction between immunology and neuroscience have revealed the reflex neural circuit mechanisms that regulate both innate and adaptive immunity [17,18]. One such well-characterized reflex circuit, termed the “inflammatory reflex”, is defined by the signals that transmit through the vagus nerve to inhibit the monocyte and macrophage production of TNF-α and other cytokines [17]. The vagus nerve is a mixed nerve, which exhibits anti-inflammatory properties through the activation of the hypothalamic–pituitary–adrenal axis by inducing the cholinergic anti-inflammatory pathway. Therefore, vagal nerve stimulation can be used to alleviate autoimmune disorders [9,10]. The underlying mechanism of dexmedetomidine-mediated anti-inflammation or the tolerance effect is thought to operate through a similar link between the immune and nervous systems. Xiang et al. demonstrated that the central α-2 agonist, dexmedetomidine, suppresses systemic inflammation through vagal- and α-7 nicotinic acetylcholine receptor-dependent mechanisms [19]. They found that dexmedetomidine increases the activity of the cervical vagus nerve and improves the survival in experimental endotoxemia through inhibiting the inflammatory cytokines [19]. Along these lines, we believe that the targeting of sedation-induced immunosuppression may provide a therapeutic opportunity to simultaneously inhibit delirium and promote tolerance.

Immunosuppression may also increase the susceptibility to microbial colonization. In our animal study, we followed weight and fur ruffling as the markers of overall health and did not observe any morbidity associated with the treatment regimen. Although the pharmacokinetics of dexmedetomidine are largely influenced by the liver rather than renal function [20], its administration did not worsen the hepatic or renal functions in our recipients, when compared to the subjects in the control group. However, if we do observe inappropriate effects, this may indicate the need to adjust the dose and schedule of the treatment regimen. Conversely, extending or increasing the administration of dexmedetomidine may significantly reduce reperfusion injury and avoid rejection in human LT.

The long-term use of immunosuppressants leads to a broad range of comorbidities. Such regimens markedly increase the susceptibility to infections and block the induction of tolerance. As such, new regimens or adjuvants devoid of these side effects that promote immunologic tolerance are always considered superior [21,22]. However, in our model, it was not sufficient to induce tolerance. To improve this effect, in our future work, we aim to strategically include tolerance-inducing drugs, for example, mTOR inhibitor rapamycin or costimulatory blockade, in combination with dexmedetomidine to promote long-term allograft acceptance [23].

This study has some limitations. First, the retrospective design in a single center restricted the sample size, and protocol bias may arise. Second, there might be a practical gap between basic research and clinical application. Unlike solid organ transplantation, mechanisms regulating ischemia–reperfusion injury in vascularized composite allotransplantation, such as the skin, are not well elucidated. In addition, their unique immunological structures indicate that the comparability of the two models presented may not be pertinent. Therefore, the influence of dexmedetomidine on effector T-cell function and differentiation needs to be further investigated and addressed in the future. Larger and prospective human studies are required to confirm these results.

## 4. Materials and Methods

### 4.1. Human Liver Transplantation

#### 4.1.1. Data Collection of Human Liver Transplantation

The clinical data, including age, sex, model for end-stage liver disease scores, causes of LT, postoperative liver function, reduction rates in hepatic transaminase (including AST and ALT) on POD 2 and 4, physical restraint time, length of stay in the ICU, and overall survival were compared between the two groups. The reduction rate of the transaminase level was calculated as follows: {(transaminase level on POD1-transaminase level on POD2)/transaminase level on POD1} × 100% and {(transaminase level on POD1-transaminase level on POD4)/transaminase level on POD1} × 100%. Higher reduction rates represented fewer liver allograft injuries. The protocol of this retrospective study was approved by the Ethics Committee and IRB of Chang Gung Memorial Hospital (approval no. 201900976B0) and conformed to the ethical guidelines of the 1975 Declaration of Helsinki.

#### 4.1.2. Immunosuppression Protocol and Surgical Techniques in Liver Transplantation

For both groups, regular immunosuppression using tacrolimus, steroids, and mycophenolate mofetil was prescribed. Methylprednisolone administration was initiated immediately after LT. Tacrolimus was introduced and adjusted according to the morning trough level on POD 1. Mycophenolate mofetil was administered from POD 14–21 (daily doses of 2 g). The details of the immunosuppression protocol and standard surgical techniques used in this study have been described previously [24,25,26]. We do not routinely remove the spleen and preserve the middle hepatic vein for donors.

### 4.2. Animal Model

Male C57BL/6 (Thy1.2^+^, H-2^b^) and BALB/c (H-2^d^) mice of 6 to 8 weeks old were purchased from the Animal Laboratory of National Institute, Taipei, Taiwan. The mice were maintained in the pathogen-free facility and were kept in accordance with the guidelines of the Animal Care Committee. Animals had free access to natural ingredient diets and water in common cages. In this non-blinded study, mice were randomly assigned to different treatment conditions. The use of these mice for experimental purposes was approved by the Chang Gung Memorial Hospital Animal Care and Use Committee.

### 4.3. Cell Culture, T-Cell Activation, and Cytokine Production

To investigate the role of dexmedetomidine in T-cell activation, naïve CD4^+^ T cells were stimulated with anti-CD3/CD28 in the medium with different doses of dexmedetomidine for 48 h. Cells were cultured in 45% RPMI 1640 and 45% Eagle Hank’s amino acid medium supplemented with 10% fetal bovine serum and antibiotics. For proliferation studies, T cells were labeled with CFSE. Dexmedetomidine was purchased from Pfizer (Brooklyn, NY, USA).

### 4.4. Flow Cytometry and Intracellular Cytokine Staining

Flow cytometry data were acquired using the BD FACS Calibur (New Jersey, USA). Cells were surface-stained. Gates were determined appropriately using unstimulated control cells. Voltage was determined using unstained controls. TNF-α concentration in the supernatant of cell cultures was analyzed using ELISA, according to the manufacturer’s instructions. Cell viability was determined via propidium iodide and annexin V staining.

### 4.5. Murine Skin Transplantation Model

Full-thickness skin grafts were harvested from BALB/c mice and transplanted onto the thoracic flank of recipient C57BL/6 mice with simple separate stitches. The size of the transplanted grafts was 1 × 1 cm^2^. The grafts were monitored daily and were considered as rejected when more than 90% of the graft tissue turned necrotic [27]. The full-thickness skin transplantation was performed under the following conditions: 1. No treatment group: positive control for rapid rejection. 2. Dexmedetomidine group: to determine if this agent exerts any effects in terms of promoting graft survival. We compared the median survival time of skin grafts in each group.

### 4.6. Statistical Considerations and Analysis

Significant differences between means were determined using an unpaired Student’s *t*-test, two-way analysis of variance, and log-rank analysis. The independent Student’s t-test was used to determine the relationships between continuous variables (expressed as mean ± standard deviation), while Pearson’s Chi-squared test was used for categorical variables (numbers and percentages). The Mann–Whitney U test was used to analyze the non-parametric data. Survival comparisons were performed using the Kaplan–Meier method with the log-rank test. All *p*-values were two-sided, and a level *p* ≤ 0.05 was considered statistically significant. All analyses were performed using SPSS software (version 24.0; SPSS Inc., Chicago, IL, USA).

## 5. Conclusions

Conclusively, the main finding of this study is that the targeting of sedation-based immunosuppression is an effective approach to transplantation. This light-sedation approach will not only make the recipients more cooperative and calmer in the ICU, but also protect allografts from injury and rejection. The immunosuppression effects of such sedatives will play a critical role in the field of transplantation, and their use may be designed toward therapeutic manipulation of the immune response. These results highlight new strategies for potentially inducing long-term transplant tolerance and survival.

## Figures and Tables

**Table 1:** The comparisons of demographic and in-hospital characteristics between the two groups.

Factors	Dexmedetomidine *n* = 23	Control *n* = 26	*p*-Value
General information			
Recipient age, median	53.0 (8.0)	58.5 (15.0)	0.179
Recipient gender (male) (%)	16 (69.6)	15 (57.7)	0.390
Donor age, median	31.0 (20.0)	28.0 (10.0)	0.527
Donor gender (male) (%)	10 (43.5)	9 (34.6)	0.525
Right lobe (%)	22 (95.7)	24 (92.3)	>0.999
MELD score, median	14 (8)	16 (13)	0.166
HBV infection (%)	13 (56.5)	10 (38.5)	0.206
HCV infection (%)	3 (13.0)	10 (38.5)	0.044
Alcohol use (%)	5 (21.7)	9 (34.6)	0.319
HCC (%)	9 (39.1)	9 (34.6)	0.744
Ascites (mL), median	350.0 (3200.0)	950.0 (2900.0)	0.379
GRWR (%), median	0.8 (0.5)	0.9 (0.5)	0.726
Clinical outcomes			
AST POD2 reduction (%), median	32.75 (4.23)	29.74 (6.44)	0.873
ALT POD2 reduction (%), median	0.14 (0.47)	0.016 (0.01)	0.317
AST POD4 reduction (%), median	0.637 (0.045)	0.636(0.081)	0.873
ALT POD4 reduction (%), median	0.39 (0.08)	0.25 (0.01)	0.087
Acute rejection (%)	13 (56.5)	17 (68.0)	0.412
Total bilirubin (mg/dL) POD7, median	4.93 (1.45)	5.55(1.5)	0.96
INR, median	1.3 (0.036)	1.23 (0.034)	0.148
ICU stay (day), median	12.0 (4.0)	13.0 (3.0)	0.035
Physical restraint time (min), median	780.0 (273.0)	1005.0 (1348.0)	0.005

Abbreviation: AST, aspartate transaminase; ALT, alanine aminotransferase; GRWR, graft recipient weight ratio; HBV, hepatitis B virus; HCC, hepatocellular carcinoma; HCV, hepatitis C virus; ICU, intensive care unit; INR, international normalized ratio; MELD, model for end-stage liver disease; POD, postoperative day.

## Data Availability

All data are contained within the article.

## Abbreviations

AST	Aspartate aminotransferase
ALT	Alanine aminotransferase
CFSE	carboxyfluorescein succinimidyl ester
ELISA	enzyme-linked immunosorbent assay
GRWR	Graft-recipient weight ratio
HBV	Hepatitis B virus
HCC	Hepatocellular carcinoma
HCV	Hepatitis C virus
ICU	Intensive care unit
INR	International normalized ratio
LT	Liver transplantation
MELD	Model of end-stage liver disease
MST	Median survival time
OS	Overall survival
POD	Postoperative day
RASS	Richmond Agitation Sedation Scale
